# In Response to: Comment on “Acquired Tracheoesophageal Fistula after Esophageal Atresia Repair”

**DOI:** 10.4274/balkanmedj.galenos.2020.2020.8.80

**Published:** 2020-10-23

**Authors:** Özlem Boybeyi Türer, Feridun Cahit Tanyel, Tutku Soyer

**Affiliations:** 1 Department of Pediatric Surgery, Hacettepe University School of Medicine, Ankara, Turkey

## To the Editor,

We have read the comment on our report entitled “Acquired Tracheoesophageal Fistula (TEF) after Esophageal Atresia Repair” by the author Vedat Akçaer (Akçaer V. Comment on “Acquired Tracheoesophageal Fistula after Esophageal Atresia Repair”. Balkan Med J doi: 10.4274/balkanmedj.galenos.2020.2020.6.85). We found some comments valuable. We would like to share our responses and comments for this letter.

Akçaer defines tracheoesophageal fistula (TEF) as esophagotracheal or esophagopulmonary fistulas instead of standard TEF (1). Although, these definitions outline the anatomical relationship between two foregut structures, the term “acquired fistula” is commonly used for fistulas between native or replaced esophagus and airway (2,3). Smithers et al. (3) classified TEFs as congenital, recurrent, and acquired according to the etiology and anatomy. In their classification, acquired TEFs include esophagobronchial (n=6), esophagopulmonary (n=2), and gastric conduit to trachea (n=2). Akçaer (1) mentions which one as “standard TEF” is not clear. If TEF persists after operation, then it is called congenital TEF. These are either missed during initial operation or are the incomplete repaired ones. Recurrent TEFs are fistulas occurred after first successful repair in the same location of index fistula. Acquired TEFs (Acq-TEF) are new pathways between airway and esophagus and occur at different location from the original fistula sites (3). Acq-TEFs can also locate between esophageal anastomosis and pulmonary parenchyma, bronchus, or the trachea. They can be seen rarely between colon and gastric conduit along with the entire respiratory system. Therefore, true nomenclature for such fistulas is Acq-TEF.

We suggested in our previous manuscript that there was no consensus on timing and type of surgical treatment in recurrent TEF (2). Akçaer stated as a comment that end-to-end anastomosis has better results regarding the frequencies of recurrent TEFs. End to-end anastomosis can be considered as a preventive method, but not a surgical management option of recurrent TEFs. There is no consensus on surgical management of recurrent TEFs.

Finally, author comments on the endoscopic treatment of TEF, complications of muscle flaps, and the use of fibrin glue in the treatment of recurrent TEFs. The fistula tract in Acq-TEFs has mostly irregular pathway and hardly localized. It is usually impossible to localize or catheterize the fistula orifice through endoscope, although minimally invasive techniques are preferred. The surgical treatment of recurrent and Acq-TEFs is more difficult and hazardous than congenital ones because of dense adhesions and mediastinal fibrosis. Failure rates are common, and several fistula repairs may be needed. Therefore, minimal invasive techniques should be kept in mind as alternative management methods (4,5). However, there is limited information and no randomized controlled trial on the use of endoscopic treatment and fibrin glue in the treatment of recurrent TEFs. The use of muscle flaps is well known and considered as a widely accepted maneuver to prevent TEF recurrence. Thus, it is difficult to suggest these treatment modalities as standards for treatment of recurrent TEFs.
